# The change in haemoglobin concentration between the first and third trimesters of pregnancy: a population study

**DOI:** 10.1186/s12884-019-2495-0

**Published:** 2019-10-16

**Authors:** David Churchill, Manisha Nair, Simon J. Stanworth, Marian Knight

**Affiliations:** 1 The Royal Wolverhampton Hospital NHS Trust, New Cross Hospital, Wolverhampton, WV10 0QP UK; 2 National Perinatal Epidemiology Unit (NPEU), Nuffield Department of Population Health, University of Oxford, Richard Doll Building, Old Road Campus, Headington, Oxford, OX3 7LF UK; 3 Department of Haematology, NHS Blood and Transplant/Oxford University Hospitals NHS Foundation Trust John Radcliffe Hospital, Oxford, UK; 4 Radcliffe Department of Medicine, University of Oxford, and Oxford BRC Haematology Theme, Oxford, UK

**Keywords:** Haemoglobin, Concentration, Pregnancy, Anaemia

## Abstract

**Background:**

The physiological fall in haemoglobin concentration from the 1st to the 3rd trimester of pregnancy is often quoted as 5 g/L. However, other studies have suggested varying levels of fall between 8 and 13 g/L. We evaluated the change in haemoglobin concentration between the 1st and 3rd trimesters of pregnancy in a multi-ethnic population of pregnant women.

**Methods:**

A retrospective cohort analysis of 7054 women with singleton pregnancies, giving birth during 2013–15 in a single urban maternity unit in England.

We calculated the changes in haemoglobin concentration from 1st to 3rd trimester using the first trimester haemoglobin as the reference point. The population was stratified into sub-groups to explore any differences that existed within the population.

**Results:**

In general the fall in haemoglobin concentration was in the order of 14 g/L or 11% of the first trimester value. This fall was consistent for the majority of sub-groups of the population. The fall was lower (7.7%) in the most deprived section of the population, IMD1, but it increased to 11.7% when we restricted that sub-group to pregnant women without health problems during the index pregnancy. Conversely, there was an increase in haemoglobin of 10.2% in women whose first trimester haemoglobin concentration was in the lowest 5% of the total study population. The population fall in haemoglobin was 10.2 g/L (7.8%), after excluding cases above the 95th and below the 5th centiles, and women with a medical and/or obstetric disorder during the pregnancy.

**Conclusion:**

The fall in haemoglobin during pregnancy is in the order of 14 g/L or 11% of the first trimester level. This is 2 to 3 times higher than suggested by some guidelines and higher than previously published work. The results challenge the current accepted thresholds for practice, and have broader implications for diagnosis and managment of antenatal anaemia.

**Tweetable abstract:**

Fall in haemoglobin across pregnancy is around 14 g/L (11%) and significantly higher than previously stated in the pregnant population. This poses questions over currently accepted thresholds for anaemia in pregnancy.

## Background

It is commonly stated that during pregnancy, the physiological fall in haemoglobin concentration (Hb) is approximately 5 g/L. This finding has been based upon changes to the lower 5th centile of haemoglobin concentration between the first and third trimesters and established guidelines that include Hb thresholds for diagnosisng iron deficiency anaemia in pregnancy [1–3]. Some older studies have derived reference curves from other published data, while others have charted the changes and found falls in haemoglobin concentration ranging between 8 to 13 g/L. But, these studies are often limited to a highly selected small samples of pregnant women [4–10].

The pregnancy related fall in the haemoglobin concentration across gestation is in the main due to the increase in plasma volume exceeding the increase in red cell mass [11]. However, the understanding of the physiological processes that influence these changes during pregnancy are still incomplete [12, 13]. Nevertheless, the change of 5 g/L is reflected in many guidelines, which state the threshold for the diagnosis of anaemia in the second and third trimesters should be lowered from 110 g/L to 105 g/L.

Understanding the changes in haemoglobin concentration is important clinically. The current thresholds for diagnosing iron deficiency anaemia (IDA) in pregnancy attempt to account for the fall in haemoglobin. Even a diagnosis of mild anaemia, will result in treatment with iron, which itself causes significant troublesome side effects for many pregnant women [14, 15].

However, our previous work has shown a significant increase in mortality and morbidity from moderate to severe anaemia, in particular, a 3 to 5-fold increased risk of stillbirth [16, 17]. Therefore, even in high income countries, true iron deficiency anaemia in pregnancy is a significant risk factor for poor obstetric outcomes and warrants detection and effective treatment.

## Aims

The aims of this study were first, to re-evaluate the reduction in haemoglobin between the first and third trimesters in a large multi-ethnic population of pregnant women. Second, to explore any differences in the degree of fall of haemoglobin in differing sub-groups by stratifying the population.

## Methods

We conducted a retrospective cohort analysis using maternity data of all women (*n* = 7054), with singleton pregnancies receiving maternity care at The Royal Wolverhampton NHS Trust between 2013 and 2015. This multi-ethnic population in the English midlands has high levels of deprivation. Deprivation was quantified using the Department for Communities and Local Government, English Indices of Deprivation 2015; the Index of Multiple Deprivation (IMD) which combines seven domains of deprivation; income, employment, education skills and training, health and disability, crime, barriers to housing and services, and living environment.

Information on maternal haemoglobin at the first trimester ‘booking’ visit, median 10 weeks’ gestation, and the 28-week visit was extracted from the hospital pathology system and then manually paired with the woman’s corresponding maternity dataset. The single dataset was then de-identfied (anonymised) by removing all personal identifiers, names, dates of birth, hospital numbers, address and postcode.

Our study data included factors known or thought to have a significant influence on haemoglobin concentration. These were ethnicity, haemoglobinopathy genotype, BMI, maternal age; hypertensive disorders of pregnancy, antepartum haemorrhage, low socioeconomic status (measured using IMD quintiles) and other medical comorbidities such as renal diseases, diabetes, chronic essential and secondary hypertension.

Maternal records relating to problems during the index pregnancy were used to generate binary variables for antepartum haemorrhage, gestational diabetes and hypertensive disorders of pregnancy. Three binary variables were generated from the history of medical co-morbidities - pre-existing haemoglobinopathies, pre-existing diabetes mellitus and any other medical comorbidities (excluding obesity).

The total study population was then stratified into sub-groups based on i) the distribution of haemoglobin concentration in the first trimester (less than 5th centile, 5th to 95th centile, >95th centile), ii) ethnicity, iii) socioeconomic status (IMD quintiles), iv) presence of haemoglobinopathy trait, and v) body mass index (BMI). Each sub-group was then refined further to eliminate the effects of specific population characteristics that may influence the change in Hb concentration. For example, women with a pre-existing medical disorder, or who developed an obstetric disorder such as pre-eclampsia, known to be associated with reduced plasma volume expansion, or women who were anaemic in the first trimester, and women with a ferritin level < 30 micrograms/L, and < 15 micrograms/L (in two separate sub-group analyses) all are more likely to be prescribed iron preparations. Non-clinical factors may also affect the change in haemoglobin concentration e.g. socioeconomic deprivation is associated with a poor diet and chronic nutritional deficiencies of iron, vitamin B12 or folic acid.

In addition, outliers below the 5th and above the 95th centiles of Hb in the first trimester were excluded from the main study population, but were investigated as sub-groups on their own in the same way as we have described above.

### Statistical analyses

We examined the distribution of first and third trimester haemoglobin concentration in the study population and corrected skewedness by removing the outliers to ensure a normal distribution around the mean. We then calculated the mean, standard deviation, inter-quartile ranges and 5th to 95th centiles for haemoglobin concentration in the first and third trimesters. We calculated absolute and percentage change in mean haemoglobin relative to the first trimester Hb. We conducted all of the above-mentioned analyses in the total population and in each population sub-group. All analyses were performed using Stata version 15.1, SE (StataCorp, College Station, TX).

Ethics committee approval was not required as this was a secondary analysis of anonymous/de-identified and routinely available hospital data.

## Results

The fall in mean haemoglobin concentration, for the whole population, from the first to third trimester was 14.2 g/L and was consistently in the region of 14 g/L or 11% of the first trimester haemoglobin concentration in the majority of sub-groups. The exceptions were women who fell below the 5th population centile in the first trimester. They had an increase in haemoglobin concetration of 10%; women who were above the 95th centile had a fall in haemoglobin of 26.6 g/L (18%) and women falling between the 5th and 95th centiles who had no co-morbid condition a fall of 10.2 g/L (7.8%) (Table 1). Women with a haemoglobinopathy trait, alpha / beta thalassaemia or a sickle cell variant also had a lower fall in haemoglobin of 9 g/L (7.7%). Adjusting the sub-groups of women stratified by their IMD rank by quintile made no material difference to the level of fall in haemoglobion which was 13 g/L. Similarly stratifying by body mass index made no difference to the level of fall. Finally ethnicity appeared to have little effect, although Blacks (women of African or Caribbean origin) had a slightly smaller fall of 11.9 g/L (9.8%), but their were considerably fewer women than in the other ethnic groups (Table 2).

**Table 1:** Total population changes in haemoglobin concentration between the first and 3rd trimesters and stratified sub-groups by distribution of haemoglobin

	1st Trimester Hb					3rd Trimester Hb					
Sample	N	mean	SD	IQ	5/95%	N	mean	SD	IQ	5/95%	ΔHB(%)
Total Population	7054	127.9	11.2	121–134	108/143	6642	113.7	10.3	107–121	96/130	−14.2(11)
Population 5th- 95th centile	6078	127.0	7.9	122–133		5745	113.0	9.5	117–120		−14.4 (11.3)
Subgroup A ƒ	4224	130	8.8	124–136	116/144	4028	115.6	9.2	110–122	101/131	−14.4 (11.0)
Subgroup B ƒƒ	5876	129.4	8.8	123–135	115/144	5476	114.7	9.6	109–121	99/130	−14.7(11.4)
Subgroup C 5th -95th centile &^a^	1958	128.1	7.86	123–134	115/140	1855	114.2	9.4	108–121	98/129	−13.9(10.9)
Subgroup D 5th–100th centile &^a^	2103	129.4	9.18	123–136	114/144	2035	114.8	9.75	109–121	98/130	−14.6(11.3)
Subgroup E = < 5 centile &^a^	78	98.7	9.59	95–106	74/107	57	108.8	12.7	99–119	89/128	+ 10.1(10.2)
Subgroup F 5th–95th centile &^a^	1303	130	7.0	126–136	118.141	1303	118	6.2	114–123	110/130	−10.2 (7.8)
Subgroup EG > 95th centile &^a^	145	147.8	5.07	144–149	1143/158	180	121.2	11.03	114–137	103/137	−26.6(18.0)

**ΔHB(%) –** represents the absolute change in haemoglobin and the percentage change in brackets

Each subgroup represents the total population excluding the women as shown by the symbols below

^a^ Restricting the sample to pregnant women **without** the following problems during index pregnancy current pregnancy problems, pregnancy induced hypertension, Pre-term birth, antepartum haemorrhage, pre-pregnancy med problems, diabetes, hypertension, renal disease, & anaemia in 1st trimester

ƒ excludes anaemic women and women with a serum ferritin <30mcg/L in the first trimester

ƒƒexcludes anaemic women and women with a serum ferritin <15mcg/L in the first trimester

**Table 2:** Total population changes in haemoglobin concentration between the first and 3rd trimesters and stratified sub-groups by ethnicity, deprivation and body size as represented by the body mass index (BMI). ΔHB(%) – represents the absolute change in haemoglobin and the percentage change in brackets

	1st Trimester Hb					3rd Trimester Hb					
Sample	N	mean	SD	IQ	5/95%	N	mean	SD	IQ	5/95%	ΔHB(%)
Total Population	7054	127.9	11.2	121–134	108/143	6642	113.7	10.3	107–121	96/130	−14.2(11)
South Asians ^a^	1097	123.4	10.9	117–130	104/140	1042	110.8	11.13	104–119	93/128	−12.6(10.2)
Blacks ^a^	294	121.5	11.4	115–129	102/139	274	109.6	9.77	104–116	93/125	−11.9(9.8)
Mixed^a^	76	127.8	8.53	122–133	113/142	74	111.0	8.48	105–118	97/125	−16.8(13.4)
White ^a^	4405	129	10.6	123–136	112/144	4191	114.9	9.81	109–121	98/131	−14.1(10.9)
Subgroup H ^b^	5663	128	10.3	122–135	111/144	5298	114	9.8	108–121	98/130	−14(10.9)
Subgroup I ^c^	728	117	12.7	109–126	95/136	676	108	12.1	100–116	88/128	−9(7.7)
IMD Q1 (most deprived)	3957	126	11.3	120–134	107/143	3720	112.8	10.5	106–120	95/129	−9(7.7)
IMD Q2	1074	126.9	11.7	122–134	107/143	1011	113.7	10.2	108–120	96/130	−13.2(10.4)
IMD Q3	778	128	10.1	122–135	110/144	739	115	9.8	109–121	99/131	−13(10.1)
IMD Q4	741	128	10.7	122–135	111/144	695	115.5	9.7	109–122	99/131	−12.5(9.8)
IMD Q5 (least deprived)	455	128.5	10.5	123–135	114/144	438	115.7	9.2	110–121	101/131	−12.8((9.3)
IMD Q1 ^a^	1037	128.9	9.06	123–135	114/144	1008	113.8	9.79	108–121	97/129	−15.1(11.7)
IMD Q2&3 ^a^	4273	126.6	11.27	120–134	107/143	4025	112.9	10.49	106–120	95/129	−13.7(10.8)
IMD Q 4&5 ^a^	918	128.6	10.4	123–135	111/144	871	115.6	9.49	109–122	100/131	−13(10.1)
BMI Obese	1711	128.8	10.8	123–136	110/145	1604	115.8	9.8	110–122	99/132	−13(16.7)
BMI Underweight	231	124.5	10.9	117–132	107/141	222	110.5	11.5	104–117	89/128	−14(17.3)
BMI normal+overweight	4946	126.5	11.2	121–134	107/143	4678	113.1	10.3	107–120	96/129	−13.4(16.9)
IMD1 excluding obese	2832	125.8	11.4	120–133	106/142	2679	111.9	10.6	105–119	94/129	−13.9 (17.4)

Each subgroup represents the total population excluding the women as shown by the symbols below

^a^ Restricting the sample to pregnant women without the following problems during index pregnancy current pregnancy problems, pregnancy induced hypertension, Pre-term birth, antepartum haemorrhage, pre-pregnancy med problems, diabetes, hypertension, renal disease, & anaemia in 1st trimester

^b^ women without a haemoglobinopathy trait

^c^ Women with an haemoglobinopathy trait

IMD = Index of multiple deprivation

By using two thresholds of ferritin to represent true iron deficiency in pregnancy and excluding women with low first trimester ferritin levels in Subgroup A (ferritin ≤30mcg/L) and Subgroup B (ferritin ≤15mcg/L) there was still consistency with our overall results with an 11.4 and 11.7% fall in haemoglobin in the respective groups.

Notably women in the most deprived IMD quintile had a fall in haemoglobin concentration of 9 g/L (7.7%) but the change in concentration increased to 15.1 g/L (11.7%) when we excluded women with obstetric and medical co-morbidities.

## Discussion

The findings of this study indicate that the fall in haemoglobin concentration during pregnancy appears to be much greater than often described in guidelines, and is of the order of 14 g/L from the first trimester level. This result should be considered in the light of a long history of defining thresholds of haemoglobin for health and disease using selected samples of “normal” pregnant women. The definition of anaemia in pregnancy used by many was first reported by the World Health Organisation 50 years ago [3], in which the lower limit of normal for pregnancy was set at 110 g/L. Interestingly, the report recognised that although these reference standards were needed, they were also “somewhat arbitrary”. The limitations of the methods used for defining anaemia have been detailed over the years, but specific to pregnancy was the failure in the WHO system to account for the normal physiological changes of the maternal cardiovascular and haematological systems. This effect was acknowledged in a later report and by the Centre for Disease Control and Prevention in the USA, that stated the haemoglobin concentration in pregnancy could fall by 5 g/L due to plasma volume expansion [2]. Other bodies recognised this and incorporated the effect in their guidance. In the British Sociey of Haematology guidelines, the lower limit of normal for haemoglobin is stated to be 110 g/L in the first trimester, 105 g/L in the second and third trimesters and 100 g/L in the puerperium, the latter to account for the blood loss of childbirth [1].

The physiological changes that take place in the cardiovascular and haematological systems during pregnancy have been extensively researched. The increase in plasma volume from the first to the third trimester is between 1100 - 1250mls, which is an increase in the total blood volume of between 40 to 50% [18]. In contrast, technical difficulties have limited establishing more accurate measures of red cell mass changes during pregnancy [13]. It is currently estimated that the increase in iron replete women is 250mls, but in women taking iron supplements the increase can be as much as 400mls. This in itself significantly alters the haemoglobin concentration and the range of haemoglobin at different gestations when compared with women not taking iron supplements [10]. The sum effect on haematocrit from these changes is a fall from 40 to 33%, with the nadir between 24 to 32 weeks’ gestation. What is known is that the physiological changes in plasma volume and red cell mass will inevitably alter the measured concentration of haemoglobin and have to be taken into account when assessing the risk ascribed to a pregnancy from ‘anaemia’.

Our study suggests that the fall in Hb concentration from the first to the third trimester could be as much as two to three times higher than the 5 g/L fall accounted for in the guidelines when defining the lower limit of normality in pregnancy. Our data identified a mean fall in haemoglobin concentration consistently in the order of 14 g/L or 11% of the first trimester haemoglobin. It was also greater than the other studies that attempted to define reference ranges during pregnancy. Some studies derived curves from reported figures and all tended to be on small, highly selected samples of women deemed to be ‘normal’ during pregnancy [6–10].

Stratification of the population was designed to evaluate sub-groups characterised by factors known to be directly or indirectly associated with changes in the haemoglobin concentration. For example, body mass index (BMI) is also positively correlated with haemoglobin, ethnicity has effects through haemoglobinopathy traits and social deprivation is more likely to be associated with poor nutrition and/or co-existing chronic medical diseases, both risk factors for anaemia. We used the IMD as a measure of deprivation, which in it’s 7 domains also accounts for nutrition and health, but also other factors that have a direct bearing upon both of these such as income and housing etc.

The sub-groups were refined further by the removal of pregnancy complications/conditions that could lead to attenuation of the changes in plasma volume and thus the change in haemoglobin across the trimesters. Even after excluding women with pregnancy induced hypertension, pre-term birth, antepartum haemorrhage, and pre-pregnancy medical problems, diabetes, hypertension, renal disease, and anaemia in first trimester, we found remarkable consistency in the fall in haemoglobin concentration between the total population and the majority of the subgroups.

The precise reasons for different levels of fall in haemoglobin concentration for some groups of women is not known, but may have implications for clinical practice. Women in the most deprived sub-group, IMD1 may have been unduly influenced by factors such as pre-existing medical disorders. When we removed women with medical disorders, including anaemia in the first trimester as a factor, the fall in this group became larger 15.1 g/L (11.7%). Alternatively, other indirect factors could have had an influence in this group. In the most socio-economically deprived group of our population the reduced fall in haemoglobin may reflect a poor diet, and so a greater likelihood of being prescribed oral iron either prophylactically or therapeutically.

Finally, we assessed whether there was an effect on fall of haemoglobin by evaluating the differences for two common diagnostic thresholds of level of ferritin, 30mcg/L and 15mcg/L for iron-deficeincy. There was no effect on different degrees of fall in haemoglobin, which supports the view that in pregnancy, ferritin is not useful when trying to assess the level of iron deficiency that requires treatment [19].

From the population distribution, women above the 95th percentile, of the population had much greater falls in haemoglobin; 26.6 g/L(18.0%). The explanation for this finding is unclear, but it might potentially indicate some, as yet unknown, homeostatic mechanism for plasma volume expansion to ensure that the blood viscosity does not impede flow through the placental vasculature.

Body mass index (BMI) did not appear to make a difference with each of the three categories, obese, underweight and normal plus overweight all having a very similar falls in haemoglobin concentration.

The limitations of our study should be acknowledged. The data are derived from a single site and from a multi-ethnic population in the industrial heart of the English midlands. Therefore, there is the potential for some local effects that may affect the findings, although the consistency of the findings after stratification suggests that any such effect is probably limited. Some categories of our subgroups contain small number of women, although the variations around the mean are in the main consistent. Nevertheless there remains uncertainty around the findings for these sub-groups.

## Conclusion

The results of this study have several implications. They indicate that the fall in mean Hb and its distrubution from the first to third trimester of pregnancy may be nearly 3 times higher (14 g/L), than the currently quoted figure of 5 g/L, which in turn determines the second and third trimester threshold for the diagnosis of anaemia during pregnancy. Our findings therefore, question the level of the lower limit of normal for haemoglobin for the diagnosis of iron deficiency anaemia in pregnancy.

There is a broad literature on the level at which the haemoglobin concentration indicates increased risk of adverse obstetric outcome. But one large population study from London suggested that ‘mild anaemia’, between 9 and 11 g/L had the lowest rate of perinatal mortality [20]. However, perinatal mortality is only one of many adverse outcomes both maternal and fetal, that are associated with anaemia. Applying a threshold approach based upon an expected quoted fall of 5g/L, as reported in some guidelines, may be an over-simplification. Our earlier published work has also shown an inverse linear relationship between the level of haemoglobin in the first trimester and stillbirth [16]. Taken in conjunction with the findings of this study, there is a need for robust epidemiological data matching the level of haemoglobin to outcome for both the fetus and mother so that appropriate advice to women on the prevention or treatment of anaemia can then be offered with more precision and confidence.

## Data Availability

The datasets generated and analysed during the current study are not publicly available due to information governance regulations, but can be made available from the corresponding author on reasonable request.
